# Supplementary material for: Dysplastic lung repair fosters a tuberculosis-promoting microenvironment through maladaptive macrophage polarization
Source: PLoS Pathog. 2025 Oct 6;21(10):e1013563. doi: 10.1371/journal.ppat.1013563 (PMC12510645; doi:10.1371/journal.ppat.1013563)
Supplement: S1 Table — (DOCX) [file ppat.1013563.s009.docx]

**S1 Table. Histopathology and acid-fast bacilli load in lung of all animals.**

| **Time post infection** | **Genotypes** | **WNL (AFB^2^ in lung)** | **mononuclear infiltrates^1^ (AFB^2^ in lung)** | **Granulomatous pneumonia only (AFB^2^ in lung)** | **Necrosuppurative pneumonia**  **(AFB^2^ in lung)** | **AFB^2^ lung** | | | | |
| --- | --- | --- | --- | --- | --- | --- | --- | --- | --- | --- |
| 11 weeks | C57BL/6J | 1/4 (-, 1/1) | 1/4 (-, 1/1) | 2/4 minimal to moderate (-, 1/2; +, 1/2) | 0/4 | 3/4 | 1/4 | 0/4 | 0/4 | 0/4 |
|  | B6.Sst1.S and B6.Sst1.S,ifnb-YFP | 0/14 | 1/14 (-, 1/1) | 10/14 mild to marked^3^ (-, 1/10; +, 3/10; ++, 3/10, +++, 2/10; ++++ 1/10) | 3/14 (++++, 3/3) | 2/14 | 3/14 | 3/14 | 2/14 | 4/14 |
| 20 weeks^4^ | C57BL/6J | 0/5 | 0/5 | 5/5 mild to moderate (+, 5/5) | 0/5 | 0/5 | 5/5 | 0/5 | 0/5 | 0/5 |
|  | B6.Sst1.S and B6.Sst1.S,ifnb-YFP | 0/15 | 0/15 | 13/15 minimal to marked^3^ (+, 4/13; ++, 7/13; +++, 2/13) | 2/15 (++++, 2/2) | 0/15 | 4/15 | 7/15 | 2/15 | 2/15 |

^1^ peribronchiolar, perivascular and/or interstitial

^2^ AFB: acid-fast bacilli. See material and method for semi-quantification criteria.

^3^ with occasional necrosis, occasional to frequent cholesterol cleft and neutrophil influx

^4^ The animals in the 20 week group have a range of time post inoculation from 19 weeks, 20 weeks, 24 weeks and 26 weeks.

C57BL/6J infected animals developed granulomatous pneumonia that affected 0.59% to 5.58% of total examined pulmonary parenchyma, with no detectable to low (- to +) Mtb load. The Mtb load in B6.Sst1.S mice was increased compared to C57BL/6J mice, with the majority ranging from ++ to ++++ (n=20). Compared to the ++++ Mtb load in the animals that developed necrosuppurative pneumonia, B6.Sst1S mice with granulomatous pneumonia displayed a maximum +++ Mtb load, with one exception in an animal that developed marked granulomatous pneumonia, which displayed a ++++ Mtb load (**Suppl.Table 3**). Rarely, B6.Sst1.S mice had no detectable Mtb in the examined sections (n=2); and a few animals had a low (+) Mtb load (n=7).
