# Supplementary material for: Dysplastic lung repair fosters a tuberculosis-promoting microenvironment through maladaptive macrophage polarization
Source: PLoS Pathog. 2025 Oct 6;21(10):e1013563. doi: 10.1371/journal.ppat.1013563 (PMC12510645; doi:10.1371/journal.ppat.1013563)
Supplement: S2 Table — (DOCX) [file ppat.1013563.s010.docx]

**S2 Table. Histopathology and acid-fast bacilli load in spleen of examined animals.**

| **Time post infection** | **Genotypes** | **WNL**  **(AFB^1^ in spleen)** | **Microgranulomas in the white pulps (AFB^1^ in spleen)** | **AFB^1^ spleen** | | | |
| --- | --- | --- | --- | --- | --- | --- | --- |
| 11 weeks | C57BL/6J | 0/4 | 4/4 rare to small numbers  (-, 2/4; +, 2/4) | 2/4 | 2/4 | 0/4 | 0/4 |
|  | B6.Sst1.S and B6.Sst1.S,ifnb-YFP | 4/14 (-, 1/4; +, 3/4) | 10/14 rare to medium numbers (+, 3/14; ++, 4/14, +++, 3/14) | 1/14 | 6/14 | 4/14 | 3/14 |
| 20 weeks | B6.Sst1.S and B6.Sst1.S,ifnb-YFP | 0/6 | 6/6 medium to large numbers (++, 4/6; +++, 2/6) | 0/6 | 0/6 | 4/6 | 2/6 |

^1^ AFB: acid-fast bacilli.

Three to four sections of spleen from each C57BL/6J infected mice (n=4 at 11 week-post infection) and B6.Sst1.S infected mice (n=14 at 11-wpi; and n=6 at 20 week-post infection) were examined. The most consistent observation across all infected animals in animals with concurrent pulmonary lesions was the presence of rare to medium numbers of microgranulomas characterized by nodular clusters of macrophage aggregates multifocally within the white pulp (**Suppl. Fig. 2A**). Most of the spleen microgranulomas contained low to intermediate Mtb load (+ to ++) with single individualized acid-fast bacilli (**Suppl. Fig. 2A**). Five B6.Sst1.S infected mice animals that developed necrosuppurative pneumonia with high Mtb load in the lung (++++) had also high Mtb load (+++) in the spleen, likely reflecting high Mtb load in the systemic circulation (**Fig. 2D; Suppl. Table 4**). Four B6.Sst1.S infected mice had no significant histopathologic findings in the spleen. In summary, in B6.Sst1.S infected mice, there were 13 out of 14 animals at 11 wpi and 6 out of 6 animals at 20 wpi with detectable Mtb in the spleen. Two of four examined C57BL/6J infected mice had no detectable Mtb in the spleen, and the other two mice had rare individualized (+) Mtb.
