# Supplementary material for: Dysplastic lung repair fosters a tuberculosis-promoting microenvironment through maladaptive macrophage polarization
Source: PLoS Pathog. 2025 Oct 6;21(10):e1013563. doi: 10.1371/journal.ppat.1013563 (PMC12510645; doi:10.1371/journal.ppat.1013563)
Supplement: S3 Table — (DOCX) [file ppat.1013563.s011.docx]

**S3 Table. Histopathology and acid-fast bacilli load in popliteal lymph nodes of examined animals.**

| **Time post infection** | **Genotypes** | **WNL**  **(AFB^1^ in popliteal lymph node)** | **Sinus histiocytosis to microgranulomas (AFB^1^ in popliteal lymph node)** | **AFB^1^ popliteal lymph node** | | | |
| --- | --- | --- | --- | --- | --- | --- | --- |
| 11 weeks | C57BL/6J | 0/2 | 2/2 mild to moderate (-, 1/2; +, 1/2) | 1/2 | 1/2 | 0/2 | 0/2 |
|  | B6.Sst1.S and B6.Sst1.S,ifnb-YFP | 1/9 (-, 1/1) | 8/9 mild to marked (-, 2/8; +, 1/8; ++, 5/8) | 3/9 | 1/9 | 5/9 | 0/9 |
| 20 weeks | B6.Sst1.S and B6.Sst1.S,ifnb-YFP | 0/6 | 6/6 medium to large numbers (++, 4/6; +++, 2/6) | 0/6 | 0/6 | 4/6 | 2/6 |

^1^ AFB: acid-fast bacilli.

In the popliteal lymph nodes, at 11 week post infection, there was mild to moderate sinus histiocytosis, with sporadic microgranulomas in the peritrabecular and medullary sinuses. Among nine B6.Sst1.S infected animals with popliteal lymph nodes available for examination, six (66.7%) contained rare to low numbers of single, acid-fast bacilli (+ to ++) in the microgranulomas; and in two infected C57BL/6J mice, one had no detectable Mtb in the popliteal lymph node; while the other one had rare (+) individual acid-fast bacilli.
