# Supplementary material for: Dysplastic lung repair fosters a tuberculosis-promoting microenvironment through maladaptive macrophage polarization
Source: PLoS Pathog. 2025 Oct 6;21(10):e1013563. doi: 10.1371/journal.ppat.1013563 (PMC12510645; doi:10.1371/journal.ppat.1013563)

**S5 Table. Pathways upregulated in multibacillary vs paucibacillary TB lesions (Total ROIs).**

MSigDB Hallmark 2020


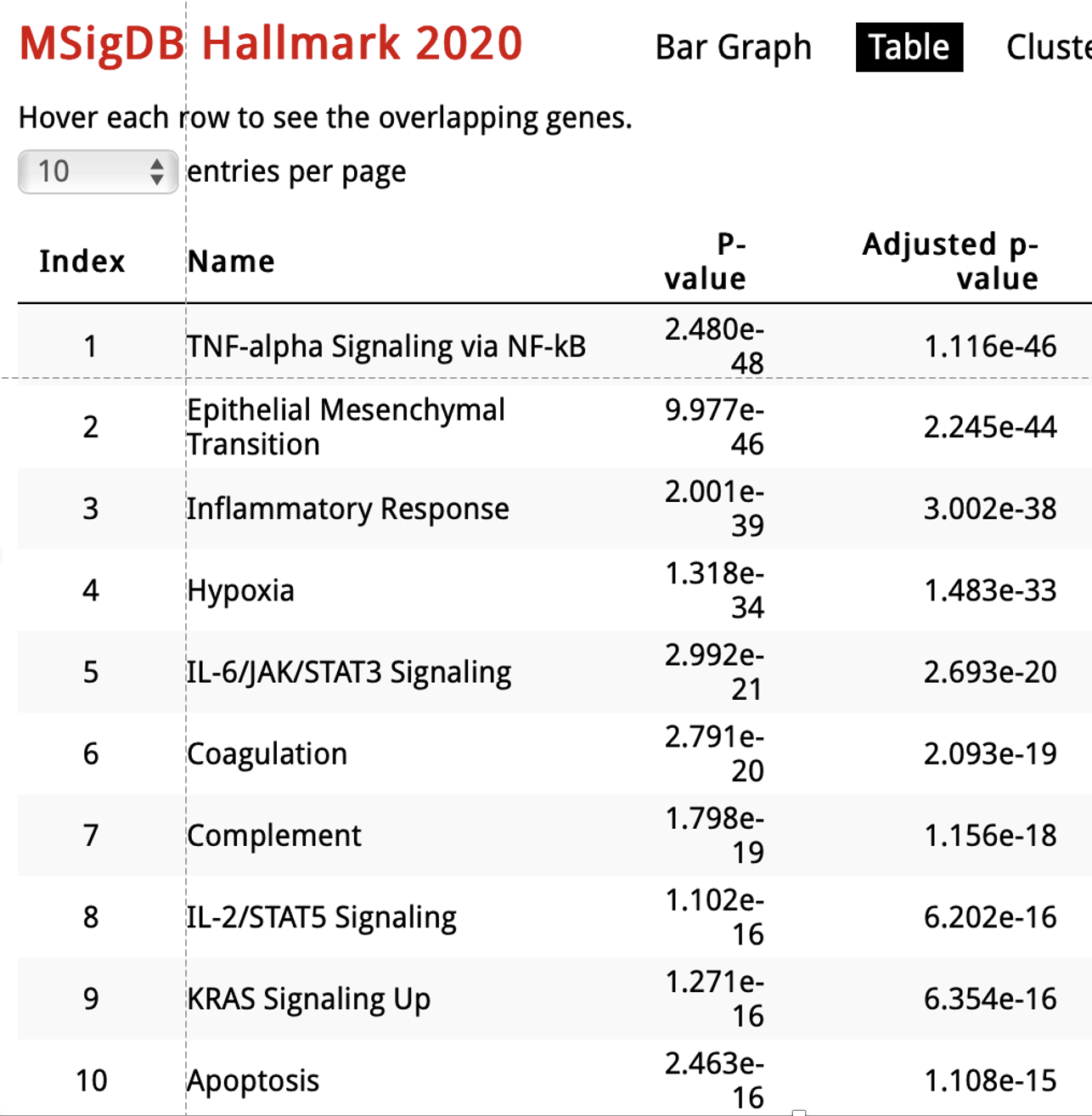

Supplement: S5 Table — (DOCX) [file ppat.1013563.s013.docx]
