# Supplementary material for: Dysplastic lung repair fosters a tuberculosis-promoting microenvironment through maladaptive macrophage polarization
Source: PLoS Pathog. 2025 Oct 6;21(10):e1013563. doi: 10.1371/journal.ppat.1013563 (PMC12510645; doi:10.1371/journal.ppat.1013563)
Supplement: S6 Table — (DOCX) [file ppat.1013563.s014.docx]

**S6 Table. The list of antibodies used for staining cells for flow cytometry.**

| **Antibody Name** | **Source: Catalog number** |
| --- | --- |
| Anti-CD11b BUV395 | BD Biosciences: 565976 |
| Anti-Ly6C eFluor 450 | Thermo Scientific: 50-246-021 |
| Anti-Sca-1 RB780 | BD Biosciences: 569230 |
| Anti-Ly6G Alexa Fluor 700 | Biolegend: 127621 |
| Anti-CD19 BV605 | Biolegend: 115539 |
| Anti-CD45 Spark Blue 574 | Biolegend: 103183 |
| Anti- Arg1 APC | Thermo Scientific: 17-369-780 |
