# Supplementary material for: Dysplastic lung repair fosters a tuberculosis-promoting microenvironment through maladaptive macrophage polarization
Source: PLoS Pathog. 2025 Oct 6;21(10):e1013563. doi: 10.1371/journal.ppat.1013563 (PMC12510645; doi:10.1371/journal.ppat.1013563)
Supplement: S7 Table — (DOCX) [file ppat.1013563.s015.docx]

**S7 Table. Cell counts of Arg1 expressing cells in bone marrow**

|  | **CD45+Arg+** | **CD11b+Ly6C+** | **CD11b-Ly6C-Sca1+** |
| --- | --- | --- | --- |
| **Uninfected mice** | 59 | 8 | 26 |
|  | 54 | 7 | 27 |
|  | 35 | 1 | 19 |
|  | 45 | 4 | 21 |
|  | 27 | 6 | 13 |
| Average | 44 | 5.2 | 21.2 |
| **Infected mice** | 160 | 45 | 62 |
|  | 174 | 22 | 123 |
|  | 119 | 65 | 33 |
|  | 282 | 44 | 174 |
|  | 100 | 25 | 57 |
| Average | 167 | 40.2 | 89.8 |
