# Supplementary material for: Dysplastic lung repair fosters a tuberculosis-promoting microenvironment through maladaptive macrophage polarization
Source: PLoS Pathog. 2025 Oct 6;21(10):e1013563. doi: 10.1371/journal.ppat.1013563 (PMC12510645; doi:10.1371/journal.ppat.1013563)
Supplement: Attachment — Submitted filename: Response to reviewers.docx [file ppat.1013563.s016.docx]

**Dear Editors,**

We appreciate the opportunity to submit the revised manuscript by Yabaji et al. “Dysplastic lung repair fosters a tuberculosis-promoting microenvironment through maladaptive macrophage polarization”. We are grateful to the reviewers for their thorough reviews and constructive suggestions for the improvement of the manuscript. In particular, both reviewers appreciated the novelty and importance of our findings for understanding TB pathogenesis, and their suggestions were primarily concerned with the clarity of presentation and technical details. We thoroughly addressed every major and minor comment in the revised manuscript.

Major concerns of Reviewer 1:

**1.** How much of the observed pathology is related to the dose of Mtb given, and what are the implications of this for translational relevance? In other published studies of infection with 10^4 Mtb in B6 mice (Kupz et al. 2016, Nemeth et al. 2020), there is little to no CFU present in the lung. This is in contrast to these findings showing scattered lesions with 10^6 Mtb. Additionally, infection with 10^4 Mtb in C3HeB/FeJ (a different genotype, albeit with similar pathology) mice did not show necrotic lesions (39007152), in contrast to these findings in B6.Sst1S mice. Could it be that this high dose in the dermis leads to rapid hematogenous dissemination prior to the onset of adaptive immunity (at least in some mice)? In such mice, lesion necrosis could occur prior to local adaptive immunity. This is critical to the authors’ conclusions and interpretation of what this model reflects. The authors should consider these possibilities in their model and discussion. A time course showing the kinetics of infection over time may shed more light onto this physiology.

**Authors:** We appreciate this cautionary note. In response we refer to our recent publication of the detailed protocol that contains information about effects of mouse genetic background, doses of infection, sex and mouse age on pulmonary TB progression in our mouse model: Yabaji, S. M. *et al.* Protocol for developing a mouse model of post-primary pulmonary tuberculosis after hematogenous spread in native lungs and lung implants. *STAR Protoc.* **6**, 103984 (2025). This reference was added to the Results and Methods sections.

In this paper Table I demonstrates that after 12 weeks post infection 10^4 CFU of Mtb Erdman induced no lung lesions in B6.Sst1S mice, 10^5 CFU induced lesions in 50% of mice and 10^6 CFU induced granulomatous PTB lesions in 100% of mice. At this stage (12 wpi) the necrotic PTB lesions were found very rarely. As shown in Table 4, the C3HeB/FeJ mice infected in hock with 10^6 CFU of Mtb Erdman survived for more than 20 weeks. In our previous studies these mice succumbed to the intravenous infection of with the same or 10X lower dose of Mtb Erdman within 4- 5 weeks. Thus, their survival and lung pathology after the hock infection, resembled the outcomes of the immune C3HeB/FeJ mice described recently (Gern, B. H. *et al.* Early and opposing neutrophil and CD4 T cell responses shape pulmonary tuberculosis pathology (2025) *J. Exp. Med.* **222**, e20250161).

We studied TB lesions of the hock-infected B6.Sst1S mice from 6 to 24 wpi. At 6 wpi the bacteria were found in regional (popliteal) lymph nodes (**Supplemental Fig.2A**), but not in the lungs. As shown in Suppl.Table 1, necrosupprative pneumonia developed in 3 out of 14 mice at 11 wpi and in 2 out of 15 mice at 20 wpi. Other mice developed moderate granulomatous pneumonia during this period of observation.

Taken together these data demonstrate that the development of individual PTB lesions in our model occurs 6 – 12 wpi, i.e. after the induction of adaptive immunity. Also it is a relatively rare event – assuming that single Mtb can give rise to a lesions, it is less than 0.0001% of the inoculum that establishes PTB lesions.

**2.** Given the lesion heterogeneity that is well described (largely by careful analysis by many of these authors) in B6.Sst1S mice, and the uncertain timing of infection within the lung. How can the authors be certain that pathology a “stepwise progression”, and smaller “incipient” lesions are earlier lesions, followed by larger lesions? To the above point, could the lesion morphology be influenced by when the lung was seeded with Mtb? From what is shown, inferences can be made about lesion size and bacterial burden, but it is difficult to support the different stage of granuloma development proposed in lines 392-400. The above suggested time course may shed some light onto this question.

**Authors:** The heterogeneity of TB lesions has been widely acknowledged in clinical experience and highlighted in recent experimental studies. In our model the development of pulmonary TB lesions is not synchronized, i.e. heterogeneous between the animals and within individual animals at the same timepoint. Therefore, we performed a lesion stratification where individual lesions were classified by a certified veterinary pathologist in a blinded manner based on their morphology (H&E) and acid-fast staining of the bacteria. Currently it is impossible to monitor progression of individual lesions in mice in real time. However, in mice TB is a progressive disease and no healing and recovery from the disease have been observed in our studies or reported in literature. Therefore, we assumed that paucibacillary lesions preceded the multibacillary ones, and not vice versa, thus reflecting the disease progression. In our opinion, this conclusion most likely reflects the natural course of the disease. However, we edited the text : *instead of disease progression* we refer to “paucibacillary” and “multibacillary” or “advanced” lesions.

The corresponding text in the Discussion was modified to remove “lesion progression” as follows: “the paucibacillary lung lesions that developed after several weeks post primary hock infection were the sites of active immune responses. These [multibacillary] lesions are shaped by inflammatory mediators associated with monocyte/macrophage lineages and, possibly, secreted bacterial products, but not an active bacterial replication or neutrophil recruitment”.

**3.** There is not enough information on number of replicates to fully evaluate this manuscript. We’re these findings repeated? Also, given the heterogeneity of lesion structures if would be helpful to know how sections were selected for downstream processing. Lastly, how many sections were analyzed per mouse, was there concordance between different sections in the same mouse.

**Authors**: The infection experiments comparing B6 and B6.Sst1S mice were performed twice. B6.Sst1S mice were further infected in three additional independent experiments, for a total of five independent infection experiments. Mice with lung and spleen implants were generated in two batches and infected in two separate experiments, with 10 implants processed and analyzed in total. One section was analyzed per mouse. In the revised manuscript, we have added details of the number of mice, lesions, and independent experiments in the corresponding figure legends to improve transparency.”

**4.** To this end, the authors used semi-quantitative determination of AFB staining was used in most cases, while in others (Figure 3), density was used. How do these determinations compare? Density would seem to be the more objective and quantitative readout between the two. Is there a reason that the authors couldn’t apply this analysis throughout their experiments? If not, I would request that this analysis (or CFU determination, if available) be completed. Also, what was the cutoff for paucibacillary and multibacillary lesions, and how was this determined? If the authors instead used a continuous variable for Mtb density, would they have greater power to identify additional differences?

**Authors:** The reasons that semi-quantification of AFB load was applied as the primary quantification approach for majority of the tissues across cohorts are:

1. The most important reason is that digital quantification is not yet well-equipped to identify tiny, rare, single AFB in some tissues producing extremely low signals. Semi-quantitative blind assessment by an experienced certified pathologist provided more reliable results.

2. The replicating AFB usually form tight clusters of overlapping bacilli that the digital area quantification does not take into account, while the manual semi-quantification approach does. Manual annotation of the regions of interests allows excluding the background areas interfering with the digital quantification. Therefore, the semi-quantitative manual approach performed by experienced pathologist in a blinded manner is a more accurate approach.

3. Lung homogenate plating for CFU determination would not allow for in-depth characterization of individual lesions that is a focus of this study.

**4**.Lastly, Why were lesions with advanced necrosis excluded from some analyses?

**Authors:** Extensive necrosis in tissues interfere with immunolabeling of cellular markers and can lead to false positive or false negative results. Besides, our goal was to reveal processes that precede the necrotization. Admittedly, we had to assume that lesions with extensive necrosis and large bacterial loads are the terminal stages of their progression.

**5.** On lines 185-186, the claim is made that the bacterial loads in the lungs always exceeded those of other regions. However, there are a number of mice in S2E where the AFB scores for non-lung organs are equal to those of this lung. This should be accurately stated, and more clarity may be shed on this issue by a fully quantitative analysis.

**Authors:** We corrected the text to reflect the data presented in Suppl.Fig.2E more accurately as follows: “In mice with high lung Mtb burdens, lung bacterial loads always exceeded those in other organs. In mice with intermediate or low lung burdens, no other organ had higher bacterial loads than the lungs”.

**6.** The lung and spleen implantation results are intriguing, and could suggest a lung parenchyma-specific role in driving necrotic lesion formation. Could there be other potential explanations such as the efficiency of neovascularization?

**Authors**: In response to the reviewer’s comment on potential differences in neovascularization between lung and spleen implants affecting Arg1⁺ macrophage recruitment and TB susceptibility, we added a panel (**Suppl. Fig. 8D**) showing abundant blood vessels in both implant types, detected by endothelial staining for caveolin-1. This Suggest that no morphologic differences in neovascularization were observed.

**7**. Also, could the authors provide a more in depth analysis to lend insight onto the inflammatory mediators that may be driving these processes (potentially informed by the spatial transcriptomics data)? Ifnb production has been sown to inhibit alternatively activated macrophage (27849167). To this point, the transcriptional data from the bone marrow would suggest that this process does not start in the lung.

**Authors**:

We thank the reviewer for providing additional support to our findings and conclusions. We have included the reference and additional text in the Discussion to stress this point:
“Our data indicate that Arg1 expression is initiated in Sca1+ progenitors in bone marrow and Mtb-infection induces the expansion of Arg1+ CD11b+Ly6C+ monocytes committed to M2-like differentiation prior to their recruitment to PTB lesions. These findings resemble the induction of myeloid progenitors committed to M2 polarization during lung tumorigenesis{LaMarche, 2024 #70} and explain simultaneous appearance of the distinct iNOS+ and Arg1+ macrophage populations within lesions dominated by the M1-polarizing cytokines, including IFN-I{Moreira-Teixeira, 2016 #81}”.

**8**. I’m also not sure what figure 7 adds here, why is it important to show in human lungs?

**Authors**: We anticipate that some of our readers would be interested specifically in this observation and may choose to use this model in the future for the analysis of parallels between the mouse and human parenchyma.

**9.** Key details are missing from the organ implantation, and the cited reference for this in the methods section is a preprint. This information should be included in this manuscript to facilitate review. To this end, certain figure panels (Figures 1A, S2E) are duplicated in a preprint from the same authors here: <https://www.biorxiv.org/content/10.1101/2025.02.28.640830v1.full> (Figures 2A and 2C).

**Authors**: We were compelled to describe our original methodology in great detail in a separate manuscript in STAR Protocols with the hope that this model will be used by other laboratories. Figures in the Protocol paper were used to present the methodology optimization and are different from the current manuscript. Thus, Fig.1A of this manuscript compares the survival of B6 and B6.Sst1S mice, while Fig.2A in the Protocol compares the survival curves of three sst1-susceptible mouse lineages: C3HeB/FeJ, B6.Sst1S and their F1 hybrids. Similarly, Suppl.Fig.2E in this manuscript compares the bacterial loads in B6 and B6.Sst1S mice, while Fig.2C in the Protocol compares the *sst1*-susceptible mice described above.

**Responses to minor comments and a list of updates.**

**Summary of updates:**

The figure legends in the revised manuscript were updated to include the number of mice and lesions analyzed and the number of experiments.

We updated the following panels in the revised manuscript per reviewer’s comments.

**Main figures**

**Figure 1**: We included the details of number experiments and animals analyzed in the figure legends.

A. Updated the panel A as per Kaplan–Meier method. Differences between groups were assessed using the Mantel-Cox (Log-rank) test.
B. Y axis label has been changed to “% weight”.
C. Added the statistical significance.

**Figure 2.**

A. Updated the panel to show plasma cells.

**Figure 3.**

D. Reanalyzed the data using individual lesions instead of whole lung (in the previous version).
E. Enhanced the size to better visibility of gene names. The lists are also available in Suppl. Table 4.

**Figure 4.**

E. Added a panel showing % of iNos or Arg+ cells among total K67+ (proliferating) cells in the lesions.

**Figure 6.**

E. Updated the figure legend to remove spleen which is presented in Suppl.Fig.8B.

**Supplemental figures.**

**Suppl. figure 1.**

A. Updated the figure legends to include the mice strain.

**Suppl. figure 2.**

A. Updated the figure legends to include the time points.

**Suppl. figure 3.**

A. Updated the figure to show better representing panels.

**Suppl. figure 6.**

A. Updated the figure legends (Spell out SPC).

**Suppl. figure 8.**

D. Added a new panel to address reviewers comment about possible differences in neovascularization of lung vs spleen implants showing abundant blood vessels in spleen and lung implant using endothelial staining with caveolin 1.

**Suppl. Tables**

We updated footnotes in Suppl. Tables 2 and 3.

**Part III – Minor Issues: Editorial and Data Presentation Modifications**
Please use this section for editorial suggestions as well as relatively minor modifications of existing data that would enhance clarity.

Reviewer #1: Figure 1A – Is there a reason this is not shown in the form of a Kaplan meier curve?

**Author: .**We updated the panel A as per Kaplan–Meier method. Differences between groups were assessed using the Mantel-Cox (Log-rank) test.

Figure 1B – What is this reflective of? Weight gain since when? If this were at the start of infection, I would have expected all mice to have 0% weight gain at day 0.

**Author:** We changed the Y-axis as percentage weight.

Figure 1C – Significance indications should be shown here

**Author:** We applied the statistical significance and included in the revised version.

Line 243 – a decline in the monocyte to macrophage ratio is not shown directly in the figures

**Author:** We updated the text to describe the trends more accurately: **“**Consistent with fmIHC, this analysis demonstrated decreased proportions of T, B, plasma cells and monocytes, and increased proportions of macrophages and neutrophils in M-type lesions”.

A consistent format should be used to display GSEA data, with the inclusion of normalized enrichment score (Similar to figure 5D).

**Author:** The format we use for presenting the data in Suppl. Table 5 contains adjusted p-values and is easier to follow for non-computational biologists.

Figure 3E is hard to read, please enlarge text and consider grouping genes into the themes discussed in the text.

**Author:** We increased the size of heatmap for better readability of gene names.

Figure 3F – how do the authors explain the discrepancy in B cells with 3D?

**Author:** Previously, automated cell quantification in Fig.3D was performed using the whole lung. In the revised analysis, we performed lesion-specific quantification. The differences observed in the B cell population follow a pattern similar to that seen in the spatial transcriptomics data.

Fig 4C – Could the authors speculate on what the implications would be of “cloaked macrophages”? They have already been activated, since they express iNOS. Is there previous work showing the consequences of macrophage-macrophage cloaking?

**Author:** We address this exciting novel observation in the Discussion (lines 447 -449).

Fig 4E – there is a wide-spread in these values (and the values are paired within the same lesion). Is there any relation to the Mtb density?

**Author:** The lesions were classified as paucibacillary or multibacillary as described above. We only performed the semi-quantitative microscopic assessment. Fig. 4E depicts the proliferation fraction of iNos+ vs Arg1+ macrophages in multibacillary lesions. It demonstrates that among iNos+ and Arg1+ the majority of proliferating cells were iNos+. We did not observe the correlation of Arg1+Ki67+ macrophages with total bacterial loads within individual lesions.

Lines 274-281 – The text refers to proportions cells and macrophages expressing a certain marker, but only the lesion area is shown. Do the authors have data on cell proportions (e.g. the percentage of Ki67+ macrophages that are iNOS+)?

**Author:** In revised version we quantified the percentage of Ki67+ cells that are either iNos+ or Arg1+ and included shown in a new panel in Fig.4E.

**Reviewer #2:** There are minor issues in the manuscript that can be readily addressed:

1. Fig 2B. Do the lymphoid follicles contain mostly plasma cells and macrophages (as suggested in the legend)? This is related to another piece of data presented in Fig. 3F. The levels of plasma cells in the paucibacillary lesions is significantly higher compared to that in multibacillary lesions based on GeoMx spatial transcriptomics studies. It would be desirable to show the markers used to identify plasma cells, particularly in the context of differentiating plasma cells from plasmablasts. Finally, in Fig. 2A, denoting plasma cells with a sign (arrows, e.g.) would better demonstrate their presence. The authors might also refer to the plasma cell data derived from the spatial transcriptomics study (Fig.3).

**Author:** we have addressed this comment by providing new panels in Fig.2A that shows classical plasma cells at high magnification indicated by arrows. Detailed analysis of B cell subpopulations in TB lesions is beyond the scope of this manuscript and will be provided in a future publication.

2. Fig. 6E, the spleen implant images are absent but described in the figure legend.

**Author:** The spleen images were moved to Suppl.Fig.8 and the figure legend was corrected accordingly.

3. The manuscript might consider delving a bit more into the discussion concerning post-primary TB, a not well understood entity. Specifically, it would be worthwhile discussing the modified B6.Sst1S-based hematogenous model in the context of post-primary TB, which could possibly differ from the natural post-primary TB scenario, in which the lung tissues that are the locale of bacterial seeding through the bloodstream have been previously infected with M. tuberculosis.

**Author:** We thank the reviewer and included a statement that post-primary TB in humans often occurs in previously infected lungs after hematogenous spread. Classic mouse models primarily recapitulate primary TB rather than post-primary TB (lines 142 –144). We will pursue the detailed discussion of our model of post-primary TB and its relevance to human TB in a subsequent review article.

4. The figure legends. In general, the figure legends can include more details so that the materials presented can be followed more easily. For example, information that connect the targeted immune cell subsets and the antibodies used would be desirable. In addition, the reproducibility of the results presented should be described – e.g., how many times had the studies been carried out. The title of Fig. 3 is “Comparative cellular and spatial transcriptomic analysis of paucibacillary and multibacillary TB lesions in B6 and B6.Sst1S mice“. However, the labeling of the results presented in not clear with respect to their relationship to the B6 versus the B6.Sst1S groups.

**Author:** Following the reviewer’s recommendations, we included the information about the numbers of animals, lesions and experiments in figure legends. The title of Fig.3 was changed – the B6 was deleted.

5. Suppl materials. In Suppl Fig.1A, the mouse strains used were not denoted. In the Suppl Table 2 and Table 3, the superscript “2” of AFB2 was not designated. In Suppl Fig.3 C, the four images of panel C (B6 Paucibacillary) is a bit confusing – specifically, the lymphoid aggregate organization of some of the images shown, particularly the bottom one, are not obvious. But the legend (as well as the text) implies that, while the lymphoid aggregates dissolute in the B6.Sst1S multi-bacillary lesions, they are more prominent in B6 P and B6.Sst1S M tissues. More precise choice of images could clarify this issue. Suppl Fig.6 , for clarity and readibility, SPC should be spelled out.

**Author:** We added the mouse strain information to Suppl.Fig.1A legend. Footnotes to Suppl.Tables 2 and 3 were updated. We replaced one image in Suppl.Fig.3C to show another example of a B cell follicle. In Suppl.Fig.6 legend SPC was spelled out (surfactant protein C, an alveolar type II cell marker).
